# Gut Microbiota Profiling of Aflatoxin B1-Induced Rats Treated with *Lactobacillus casei* Shirota

**DOI:** 10.3390/toxins11010049

**Published:** 2019-01-17

**Authors:** Winnie-Pui-Pui Liew, Sabran Mohd-Redzwan, Leslie Thian Lung Than

**Affiliations:** 1 Department of Nutrition and Dietetics, Faculty of Medicine and Health Sciences, Universiti Putra Malaysia, 43400 Serdang, Selangor, Malaysia; liew.winnie@outlook.com; 2 Department of Medical Microbiology and Parasitology, Faculty of Medicine and Health Sciences, Universiti Putra Malaysia, 43400 Serdang, Selangor, Malaysia; leslie@upm.edu.my

**Keywords:** Aflatoxin B1, *Lactobacillus casei* Shirota, Alloprevotella, metagenomic sequencing, microbiota

## Abstract

Aflatoxin B1 (AFB1) is a ubiquitous carcinogenic food contaminant. Gut microbiota is of vital importance for the host’s health, regrettably, limited studies have reported the effects of xenobiotic toxins towards gut microbiota. Thus, the present study aims to investigate the interactions between AFB1 and the gut microbiota. Besides, an AFB1-binding microorganism, *Lactobacillus casei* Shirota (Lcs) was tested on its ability to ameliorate the changes on gut microbiota induced by AFB1. The fecal contents of three groups of rats included an untreated control group, an AFB1 group, as well as an Lcs + AFB1 group, were analyzed. Using the MiSeq platform, the PCR products of 16S rDNA gene extracted from the feces were subjected to next-generation sequencing. The alpha diversity index (Shannon) showed that the richness of communities increased significantly in the Lcs + AFB1 group compared to the control and AFB1 groups. Meanwhile, beta diversity indices demonstrated that AFB1 group significantly deviated from the control and Lcs + AFB1 groups. AFB1-exposed rats were especially high in *Alloprevotella* spp. abundance. Such alteration in the bacterial composition might give an insight on the interactions of AFB1 towards gut microbiota and how Lcs plays its role in detoxification of AFB1.

## 1. Introduction

Mycotoxins, a structurally diverse group of poisonous fungal secondary metabolites that contaminate agricultural crops during pre-harvest or post-harvest storage in the hot and humid climate regions [1]. Among the well-known mycotoxins, aflatoxin B1 (AFB1) is the most ubiquitous and poisonous mycotoxins, which has been categorized as group I carcinogen [2]. Moreover, AFB1 also causes significant economic losses of crops globally [3]. Several studies have produced conclusive evidence that the carcinogenicity of aflatoxins operates via a mutagenic mechanism. The process involves the cytochrome (CYP-450) enzyme metabolism systems, the formation of genotoxic metabolite (AFB1-8,9-epoxide), the generation of DNA adducts, as well as the alteration of tumor suppressor (TP53) gene [4]. In addition to its carcinogenic properties, AFB1 also induces a number of health problems, such as gastrointestinal (GI) pain, diarrhea, as well as affects the growth and development in both animals and human beings, as demonstrated in numerous studies reviewed by Gong et al. [5].

The negative impacts of AFB1 towards GI tract is of high concern since AFB1 commonly enter the host via food contamination [6]. Diet contaminated with AFB1 influences the GI tract, subsequently causes epithelial injuries in the stomach and intestine, primarily, intestinal inflammation in animal models included rat, pig, and chicken [7]. Both in vitro and in vivo studies have demonstrated that AFB1 induces intestinal damages via perturbation of the intestinal barrier and activation of immune system, cell apoptosis, and cell proliferation [7]. At the same time, AFB1 exposure can cause gut dysbiosis and disrupt the gut microbiota balance by increasing the growth of non-beneficial and pathogenic bacteria as discussed by Liew and Mohd-Redzwan [8]. Moreover, gut dysbiosis can affect the health condition of the host as reported in numerous studies [9].

The gastrointestinal tract is colonized by the largest community of bacterial members of the microbiota which made up of a rich variety of microorganisms [10]. Substantial progress in the gut microbiota research has discovered the vital role of gut microbiota in maintaining health status [11]. Such metagenomic studies were made possible with the improvement of currently available next-generation sequencing (NGS) technologies, which reduce the cost and increase the throughput of bases sequenced/run concurrently [12]. The involvement of gut microbiota on host physiological functions and metabolic activities, such as through the activation of the immunity, excretion of fermentation products, and inhibition of colonization by pathogens has been well recognized [13].

An altered gut microbiota composition is affected by several factors including genetics [14], stressful experiences [14], dietary changes [15], as well as the development of disorders and diseases [16]. Recently, the gut microbiota dysbiosis has frequently been associated with the development of various diseases [16]. A vast range of gut microbiota-related diseases have been discovered, such as food allergies, asthma, obesity, cardiovascular disease, diabetes, eczema, autism, irritable bowel syndrome, Crohn’s Disease, colon cancer, hepatic encephalopathy, and mental disorders [17]. A study demonstrated that metabolism products and enzymes from pathogenic microorganisms lead to higher level of carcinogenic compounds [18]. However, studies on the reactions of AFB1 towards the gut microbiota are limited.

Probiotics are well recognized for their vital roles in maintaining wellbeing, especially gut health and microbiota restoration [17]. Probiotics are defined as “live micro-organisms which, when administered in adequate amounts, confer a health benefit on the host” [19]. Among all probiotics, the most conventional bacteria used in both human and animal are lactic acid bacteria (LAB), especially *Lactobacillus* spp. [20]. *Lactobacillus* spp. have recently become the focus of health-promoting bacteria research in diarrhea, lactose intolerance, allergies, infections, cholesterol reduction, eczema, immune function, as well as central nervous system dysfunctions [21]. Additionally, some species of *Lactobacillus* have aflatoxin-reducing activities [22]. Studies revealed that 2 × 10^10^ CFU/mL of *Lactobacillus* sp. is able to reduce the AFB1 level to 0.1–13% [23]. It appears that the surface components of probiotic bacteria are involved in AFB1-binding [24]. It is worth to mention that probiotic intervention is potentiated to alleviate AFB1-induced toxicity [25].

In the present study, *Lactobacillus casei* Shirota (Lcs) was selected for the AFB1 removal purpose. Lcs is notable for its status in the healthcare industry, especially in maintaining gastrointestinal health [26]. Lcs has previously exhibited high affinity for binding AFB1 in animal [27], as well as in human [28]. Despite this, further studies are necessary in order to evaluate the possible effects of Lcs towards microbiota at the intestinal level under chronic AFB1 exposure. This research involves the investigation of the gut microbiota changes by Lcs in the AFB1 detoxification process. Such knowledge may discover novel approaches for both the treatment, as well as prevention, of mycotoxin contamination and mycotoxicosis. Predicated upon that, it is hypothesized that the gut microbiota composition of rat would be influenced by the toxic effects of AFB1. Besides, the AFB1-altered gut microbiota composition can be recovered upon Lcs treatment.

## 2. Results and Discussion

### 2.1. Sequencing and Bacterial Abundance

The filtered rRNA sequences obtained from the colon contents of the control, AFB1, and Lcs + AFB1 dietary groups, resulted in a total of 703,616 sequences, with read lengths averaging 450 nucleotides and GC contents of 53%. Among all, 218,312 sequences belonged to control feces, while 240,975 and 244,329 represented AFB1 and Lcs + AFB1 feces respectively. The number of rRNA sequences from individual control samples ranged from 49,957 to 61,517; those from AFB1-treated samples ranged from 52,567 to 69,055 while those from Lcs + AFB1-treated samples ranged from 49,161 to 74,075. Normalization was performed on the number of operational taxonomic units (OTUs) by subsampling 45,771 sequences from each individual sample.

**Figure 1:**
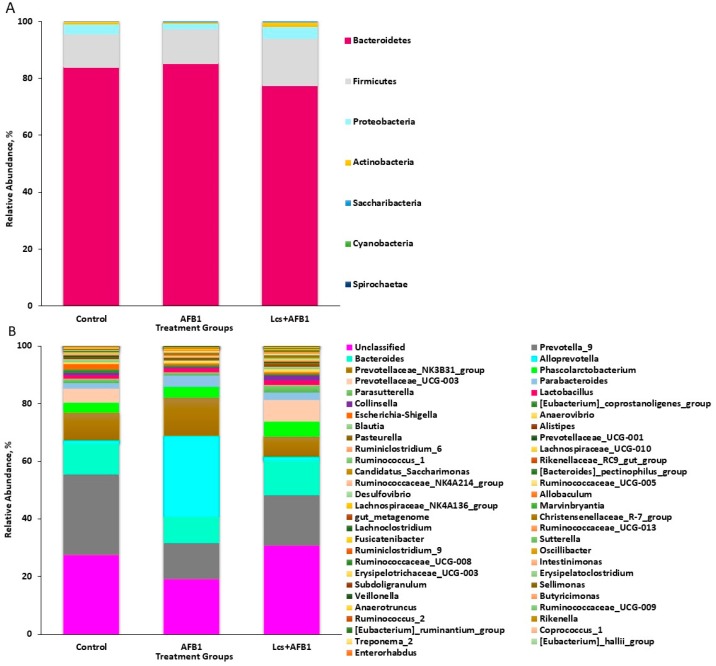
Microbial taxonomic profiles from the fecal contents of the three treatment groups at the phylum (**A**) and genus (**B**) levels, classified by the representation of >1% of the total sequences. The X-axis is the sample name or group name, and the Y-axis is the relative abundance (taxon reads/total reads in the gut microbiota) of different species. The legend is the name of the taxonomic classification of the species.

All rRNA sequences from the fecal contents were grouped into known phyla. The phyla Bacteroidetes and Firmicutes were dominant and they were represented by 81.99% and 13.51% of all rRNA sequences, respectively, as shown in Figure 1A. Proteobacteria, which was represented by 3.27% of the total sequences was the third highly abundant phylum. Some of the phyla were each represented by <2.00% of all rRNA sequences. The phyla Actinobacteria and Saccharibacteria were represented by 1.7% and 1.0% of all rRNA sequences, respectively. Whereas, the remaining phyla constituted <0.3% of the total rRNA sequences. The minorities were represented by Cyanobacteria (~0.01%) and Spirochaetae (~0.01%).

**Figure 2:**
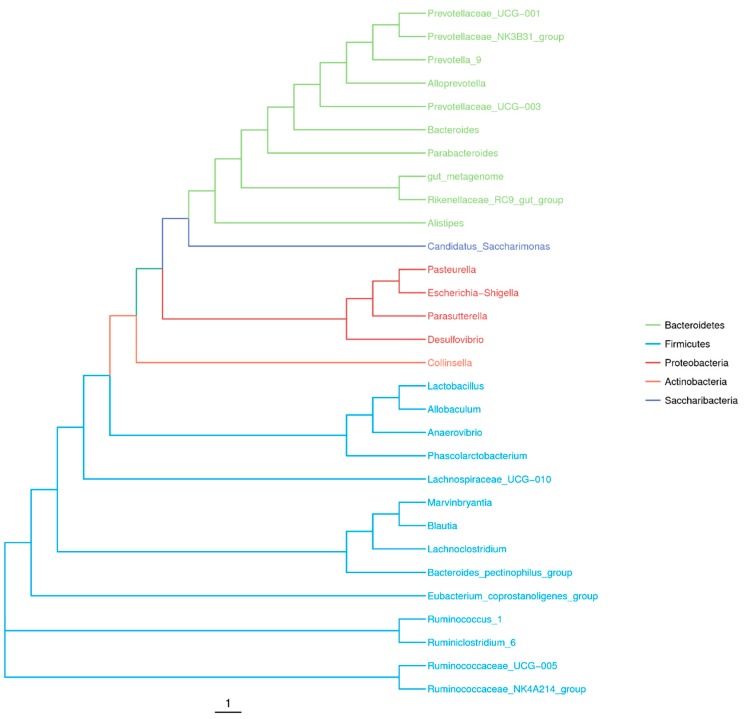
Phylogenetic tree of the top 30 most abundant operational taxonomic unit (OTU) sequences. The color of the branch indicates its corresponding phylum, different colors represents different phylum.

At the genus level (Figure 1B), Prevotella was highly abundant, comprised 19.35% (106,271) of the total rRNA sequences. There was a group of taxa showing high abundance at the phylum level, which includes Firmicutes and Bacteroidetes, but were not classified at the genus level. Meanwhile, some genera were each represented around 10% of the total sequences, such as *Bacteroides* (10.82%), *Alloprevotella* (10.05%), and *Prevotellaceae*_NK3B31_group (9.93%). Other genera, represented more than 0.5% of the total sequences, were *Phascolarctobacterium* (4.19%), *Prevotellaceae*_UCG-003 (4.03%), *Parabacteroides* (2.95%), *Parasutterella* (1.65%), *Lactobacillus* (1.36%), *Collinsella* (0.98%), *Eubacterium_coprostanoligenes*_group (0.97%), *Escherichia-Shigella* (0.88%), *Anaerovibrio* (0.85%), and *Blautia* (0.72%). A phylogenetic tree in Figure 2 infers approximately-maximum-likelihood phylogenetic relationship from the alignments of the top 30 most abundant OTU sequences.

Generally, the gut microbiota of human and laboratory animals, such as rat and mice are dominated by two major phyla, Bacteroidetes and Firmicutes [29]. As shown in this study, Bacteriodetes and Firmicutes are the dominant phyla followed by other major phyla, such as Proteobacteria, Actinobacteria, and Saccharibacteria. The fecal contents from all the treatment groups consisted of similar phyla. The phyla found in the rats’ gut microbiota are commonly reported in previous rat microbiota studies [30,31]. In this study, rats were chosen as study subjects due to rats are a better representative of the human gut microbiota compared to other animals [32]. The gut bacterial communities of rats are comparable to the gut microbiota of human. Most of the genera obtained from this study are common microbiota found in rat’s fecal content [30,31]. Based on the microbial taxonomic profiles at phylum and genus levels, a different distribution pattern can be observed. Therefore, the clustered OTUs of each sample were subjected to analysis on their alpha and beta diversity.

### 2.2. Alpha and Beta Diversity

**Figure 3:**
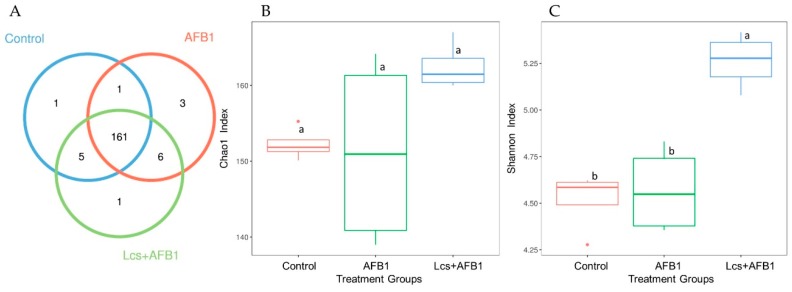
OTU Venn diagram (**A**) and alpha diversity indices (Chao1; (**B**) and Shannon index: (**C**)). The circles of different colors in the Venn diagram represent different treatment groups, and the numbers in the figure represent the numbers of OTUs unique or common to each treatment group. In the petal diagram, each petal represents a treatment group. The numbers on the petals represent the number of OTUs unique to the treatment group, and the white circle in the middle represents the number of OTUs shared by all groups. The chao1 index showed boxplot of each group. The X-axis indicates the names of the groups and Y-axis indicates the Chao 1 index. Each box diagram shows the minimum, first quartile, medium, third quartile and maximum values of the chao1 index of the corresponding treatment groups. Graph C is the Shannon index boxplot of each group. Means between different treatment groups with different superscript letters (a and b) are significantly different (*p* < 0.05).

According to the results of OTU cluster analysis using the sequences obtained, Venn diagram (Figure 3A), as well as the alpha diversity indices (Chao1 and Shannon diversity index), were analyzed (Figure 3B,C). Chao index indicates the richness of the community, the estimated number of species/features per sample; and Shannon index indicates community diversity [33]. The Chao index showed that all the treatment groups were not significantly different from each other in term of microbiota richness, although the mean value of control group and Lcs+AFB1 were higher compared to AFB1-treated group (Figure 3B). For a better understanding of the shared richness among each group, a Venn diagram was illustrated to display the overlaps between groups [34]. OTU Venn diagram plotted indicates the common and unique OTUs among the three treatment groups (control, AFB1, and Lcs + AFB1). This analysis showed that the core microbiota consisted of 161 OTUs (Figure 3A). Results showed that there were only 17 unique OTUs found. Among the three treatment groups, the microbiota of Lcs + AFB1 treated rats had significantly higher Shannon diversity index compared to control and AFB1-treated groups (Figure 3C). Furthermore, the Good’s coverage estimator is evaluated to calculate the percentage of diversity captured by the devoted sequencing effort. In this study, the average Good’s coverage was 100% for all samples, indicating that the true number of OTUs was adequately represented [35].

**Figure 4:**
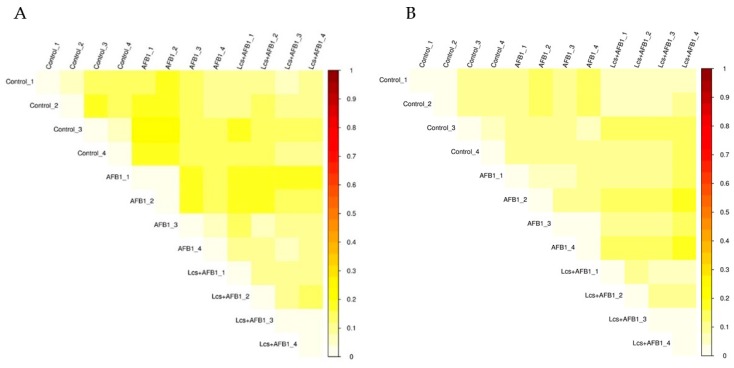
Heatmap of Unweighted Unifrac (phylogenetic distance; **A**), and Weighted Unifrac (phylogenetic distance weighted by abundance counts; **B**). The color scheme in the heatmap represents the degree of difference between the two samples. The lighter the color, the smaller the coefficient between the two samples, and the smaller the difference in species diversity.

**Figure 5:**
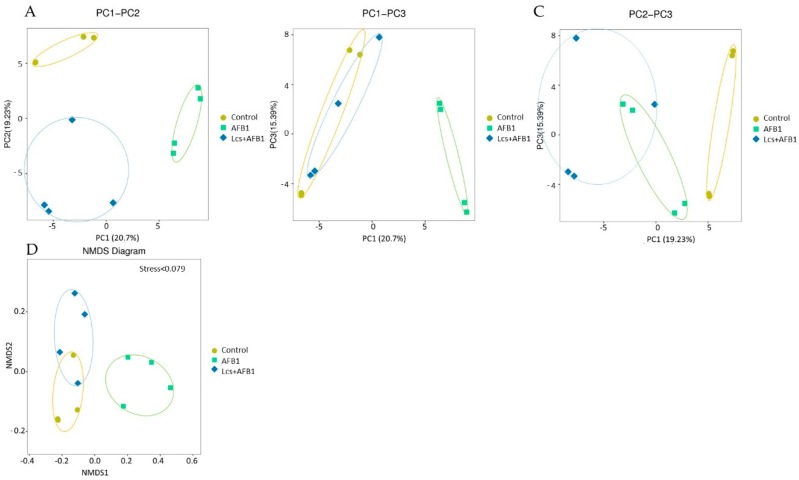
Beta diversity measures using Bray-Curtis (counts; **A–C**), and non-metric multidimensional scaling (NMDS) diagram (**D**). PC1, PC2, PC3 represent the first, second and third principal components, respectively. The percentage after the principal component represents the contribution rate of this component to sample difference and measures how much information the principal component can extract from the original data. The distance between samples indicates the similarity of the distribution of functional classifications in the sample. The closer the distance, the higher the similarity. NMDS diagram accurately reflects the difference between the samples with a stress value <0.2.

**Figure 6:**
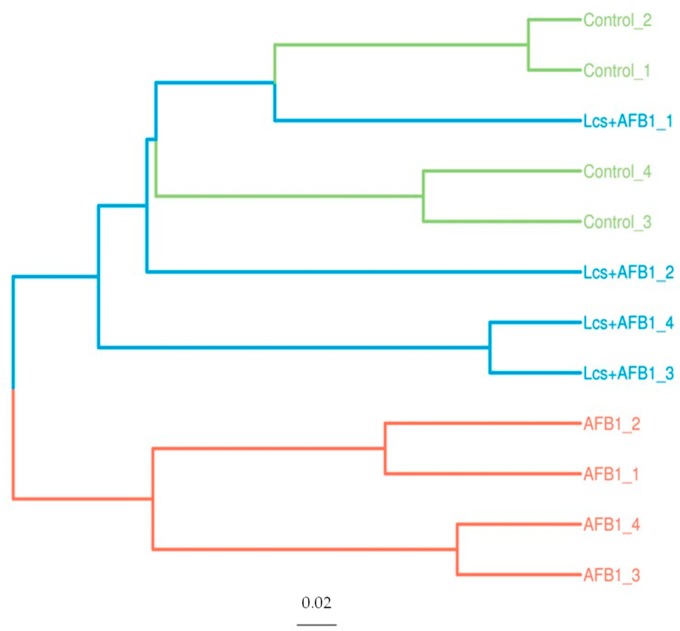
Unweighted pair group method with arithmetic mean (UPGMA)-Tree Cluster Analysis. Each branch in the figure represents a sample. Different colors representing different groups.

In order to measure the extent of similarity between microbial communities in the treatment groups, their beta diversity was calculated by unweighted/ weighted UniFrac (Figure 4), and principal coordinate analysis (PCA) (Figure 5A) was performed [36]. PCA analysis is a statistical method to determine the key variables in a multidimensional data set that is most responsible for the differences in the observations and thus is commonly used to simplify complex data analysis [37]. PCA and UniFrac distance heat maps showed that gut microbial communities in AFB1 group and Lcs + AFB1 group were different from that of untreated control rats. It was shown that gut microbiota in the control group of normal rats and in rats that were challenged by AFB1 was distributed in different regions. This result indicated that AFB1 ingestion altered the microbiota composition. Based on the beta diversity distance matrix, the non-metric multidimensional scaling (NMDS) plot showed a clear clustering of the AFB1 samples from the control and Lcs-AFB1 samples. The stress value of NMDS obtained is <0.2 (0.079) which indicates the results can accurately reflect the difference between the samples [38]. These results indicate that diet with AFB1 and Lcs, both influence mammal gut bacterial diversity. Subsequently, the differences were estimated using the unweighted pair group method with arithmetic mean (UPGMA) tree cluster analysis, which uses evolutionary information derived from sample sequences to calculate whether samples in a specific environment is significantly different from an evolutionary lineage in microbial communities [39]. The results in Figure 6 indicated that the phylogenetic relationship of the AFB1 group was relatively far from the control group. In contrast, the AFB1-induced rat which received Lcs treatment was phylogenetically close to the control group. Overall, the results from the beta diversity index demonstrated that gut microbiota in the AFB1-treated rat was normalized to the microbiota diversity of untreated rat after Lcs probiotic treatment.

### 2.3. Variation Analysis Between Groups

Analysis of similarities (ANOSIM) and linear discriminant analysis effect size (LEfSe) were used to further confirm the differences between the control, AFB1, and Lcs-AFB1 groups. The ANOSIM statistic examines the mean of ranked dissimilarities between groups to the mean of ranked dissimilarities within groups [40]. It is used to determine whether the grouping is meaningful. An R-value near to 1.0 indicates dissimilarity between groups, whereas an R-value near to 0 indicates an even distribution of high and low ranks within and between groups [41]. There is a significant separation of bacterial composition was observed between control and AFB1 group (ANOSIM *R* = 0.292; Table 1) with *p*-value < 0.05. After Lcs treatment, the difference between the control group was significantly increased to R-value of 0.87 with *p*-value < 0.094. Such dramatic changes revealed the efficiency of Lcs in removing AFB1, and thus reducing AFB1-induced microbiota changes in the rat.

**Figure 7:**
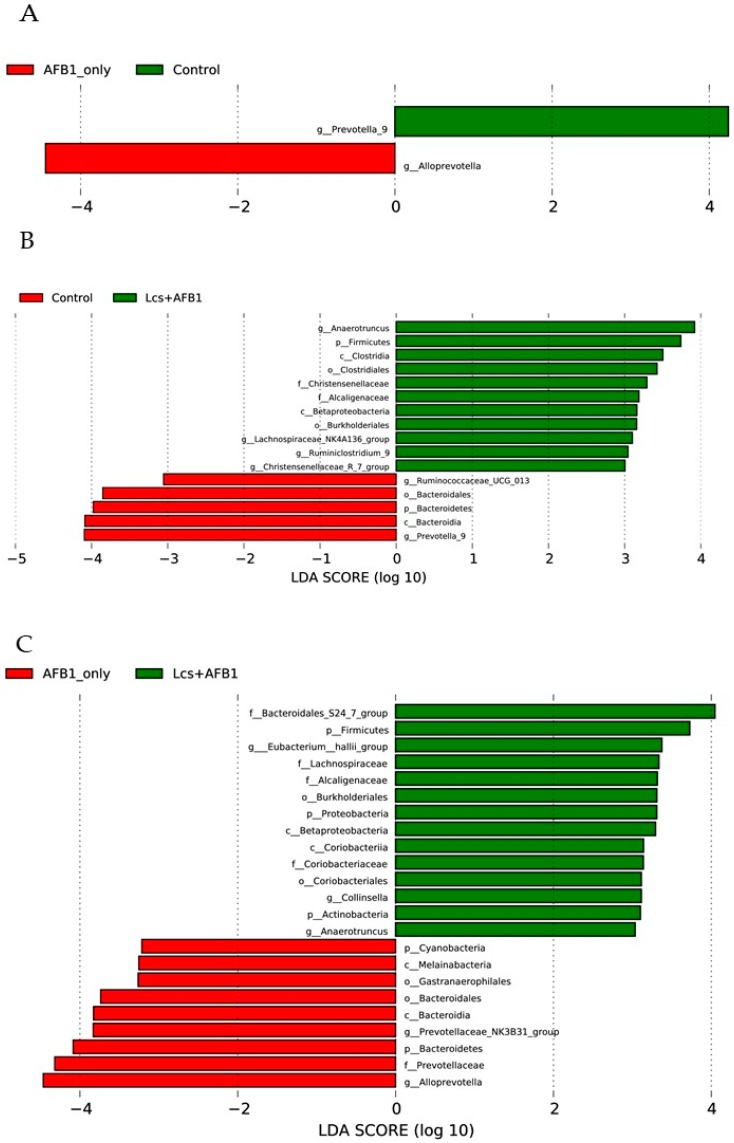
The figure shows the categories of species that are significantly different between the two groups (AFB1 group vs Control group, (**A**) Control group vs Lcs + AFB1 group, (**B**) AFB1 groupvs Lcs + AFB1 group, (**C**) as well as the LDA score from LDA analysis.

Lastly, the taxa that best characterized each population was determined using LEfSe with default parameters on species-level OTU tables [42]. In the present study, LEfSe was calculated to identify bacterial taxa differentially distributed between control, AFB1, and Lcs-AFB1 group (Figure 7A–C). The evolutionary relationships of the differential taxa in all the tested groups were plotted using cladograms (Figure S1A–C). In Figure 7A, 2 genera were differentially represented in the control and AFB1 group. Alloprevotella was found dramatically high in the AFB1 group compared to the control group. In contrast, the abundance of Prevotella_9 reduced significantly after AFB1 ingestion. It is worth to note that the distinct difference which distinguished AFB1-induced rats from the untreated control rats is the reduction of a group of unclassified microorganisms at the genus level (Figure 1B).

A total of 16 bacterial taxa were differentially represented among the control and Lcs + AFB1 groups, with 8 more abundant bacterial taxa with increasing trends (g_Christensenellaceae_R_7_group, g_Ruminiclostridium_9, g_Lachnospiraceae_NK4A136_group, o_Burkholderiales, c_Betaproteobacteria, f_Alcaligenaceae, f_Christensenellaceae, o_Clostridiales, c_Clostridia, p_Firmicutes, and g_Anaerotruncus) in the Lcs + AFB1 group. Similar to AFB1 group, the abundance of Prevotella_9 in Lcs + AFB1 group was reduced tremendously compared to the control group. Such results indicate Prevotella_9 is one of the key changes after AFB1 ingestion. Besides, c_Bacteroidia, p_Bacteroidetes, o_Bacteroidales, and g_Ruminococcaceae_UCG_013 were depleted in the Lcs+AFB1 group, in relation to the control group.

Comparing both AFB1 and Lcs + AFB1 groups, g_Anaerotruncus, p_Actinobacteria, g_Collinsella, o_Coriobacteriales, f_Coriobacteriaceae, c_Coriobacteria, c_Betaproteobacteria, p_Proteobacteria, o_Bukholderiales, f_Alcaligenaceae, f_Lachnospiraceae, g_Eubacterium_hallii_group, p_Firmicutes, f_Bacteroidales_S24_7_group were overrepresented in the Lcs + AFB1 group in an increasing order. On the other hand, p_Cyanobacteria, c_Melainabacteria, o_Gastranaerophilales, o_Bacteroidales, c_Bacteroidia, g_Prevotellaceae_NK3B31_group, p_Bacteroidetes, f_Prevotellaceae, g_Alloprevotella were depleted in the Lcs + AFB1 compared to AFB1 group.

Such bacterial composition alteration might give an insight into the interactions of AFB1 towards gut microbiota. In this study, *Alloprevotella* spp. was found present abundantly in the feces of AFB1-treated rats. *Alloprevotella* spp. belongs to the order Bacteroidales. In a study conducted by Wang et al. [43], the bacterial compositions of Bacteroidales was found significantly increased in a dose-dependent manner of AFB1. *Alloprevotella* spp. has been related to the high production of succinic acid and acetic acid as end products [44,45]. Succinic acid at a high level can cause pathological conditions includes inflammation, tissue injury and malignant transformation [46,47]. Acetic acid, on the other hand, may induce colitis at high concentration and frequently used to produce ulcerative colitis animal model [48]. These microbial products from *Alloprevotella* spp. may induce damages to the GI tract.

The current study is an extension of previous work where we found that AFB1 exerted harmful effects towards small intestine and colon [27]. In the AFB1-exposed rat, lymphocytes accumulation was observed in both small intestine and colon, moreover, large carcinoma was detected in the small intestine. Abundant accumulation of lymphocytes implies localized inflammation [49]. Similar findings have been reported in another study by Nurul Adilah et al. [50] where AFB1 exerted damaging effects towards GI tracts especially in the small intestine. The GI tract, specifically small intestine is the main absorption site of ingested aflatoxin [28]. AFB1 may induce intestinal damages directly via the generation of the genotoxic metabolite. The intestinal epithelial cells produce CYPs which convert AFB1 into the reactive epoxide and subsequently into AFB1-DNA adducts. The negative reactions on the gut from AFB1 exposure include the disruption of the intestinal barrier, cell proliferation, cell apoptosis, and immune system [51]. Indirectly, AFB1 may impair the gut health through gut microbiota perturbation as revealed in this study. Such alteration in gut microbiota profiles may lead to gut dysbiosis. Following that, gut dysbiosis may cause the disruption of intestinal barrier function and bacterial overgrowth. In this study, AFB1-induced gut dysbiosis which leads to overgrowth of *Alloprevotella* spp. A high abundance of this genus has been associated with the carcinogenic process [52]. The alteration in gut microbiota balance could be one of the pathways exploited by AFB1 to induce gut damages. AFB1 perturbates the gut microbiota balance and leads to gut dysbiosis. Without the protection from a healthy gut microbiota, AFB1 can induce intestinal inflammation and carcinoma.

Surprisingly, the increased abundance of *Alloprevotella* spp. was not observed in the Lcs + AFB1 group (Figure 7B,C). On the other hand, previous works on histopathological analysis of Lcs + AFB1 group’s intestinal tissues revealed that Lcs can alleviate the detrimental effects induced by AFB1 [27,50]. The result is correlated with this study where the overgrowth of *Alloprevotella* spp. was inhibited by Lcs. Therefore, the study demonstrated that Lcs treatment can protect the GI tracts of the studied animal against AFB1 toxicity. Probiotic Lcs has been demonstrated to protect the gut via various mechanisms. Apart from the direct removal of AFB1, Lcs also produces metabolic byproducts which offer health-promoting effects for the host [53]. Several studies also revealed that Lcs treatment can positively modulate the gut microbiota profile of the host, which eventually improves the health status of the host [54,55]. Lcs colonized the GI tracts and suppress the growth of pathogenic microorganisms via production of antimicrobial agents or competitive exclusion [56]. Moreover, Lcs can control the intestinal immunity and modulate the reactions of the intestinal epithelia and immune cells towards the microorganisms in the intestinal lumen [54]. Beside its AFB1-binding ability, Lcs may alleviate AFB1-induced toxicity via gut microbiota modulation.

Even though the current knowledge on gut microbiota is inadequate, however, their roles in maintaining and influencing the host health have been frequently reported. As mentioned previously, various important immune and metabolic disorders are known to be affected by the imbalanced gut microbiota. Therefore, it is crucial to maintaining the balance of gut microbiota for the health maintenance of the host. The findings in this study revealed that AFB1 caused substantial gut microbiota alteration. However, Lcs was able to ameliorate the gut microbiota composition alteration induced by AFB1. The results from beta diversity and UPGMA-Tree Cluster Analysis demonstrated that the Lcs treatment reversed the aberrant gut microbiota profile and shifted the gut microbiota composition of the AFB1 group to be substantially like that of the control group.

## 3. Conclusions

In conclusion, the intestinal bacterial flora was significantly affected by AFB1 and Lcs. Particularly, the AFB1 group demonstrated a high abundance of *Alloprevotella spp.* among all the groups, possibly suggesting its role in aflatoxicosis induced by AFB1 via production of short chain fatty acids (SCFAs), such as succinic acid and acetic acid. It is therefore recommended to pay attention to the concentration of SCFAs in the feces for future relevant study. Meanwhile, Lcs significantly modulated the AFB1-induced gut microbiota fluctuations back to normal level. It is suggested that these changes will eventually influence the toxic effects of the xenobiotic agent. Extensive in-depth studies are required to investigate the microbial products in the gut which may affect the AFB1 toxicity level. Such future studies may reveal potential relationships between AFB1, Lcs, and the gut microbiota, to develop an alternative therapy for aflatoxicosis occurrences.

## 4. Materials and Methods

AFB1 was acquired from Trilogy Analytical Laboratory, Inc. (Vossbrink Drive, WA, USA). The bacterial culture media were Man deRogosa (MRS) broth (Himedia, Bombay, India), MRS agar (Himedia, Bombay, India), and glycerol solution (Sigma-Aldrich, St. Louis, MO, USA). Phosphate-buffered saline was purchased from Merck (Darmstadt, Germany).

### 4.1. Ethics Statement

The use of animal in the present experiment was subjected to review and approved by the Institutional Animal Care and Use Committee of Universiti Putra Malaysia on 1 March 2017 (UPM/IACUC/AUP-R098/2016).

### 4.2. Bacterial Culture

The bacterial culture of Lcs was isolated from Yakult^®^ fermented milk product. In order to confirm the identity of the bacteria, the bacterial 16s RNA sequence was analysed by First BASE Laboratories Sdn. Bhd (Seri Kembangan, Malaysia). Using the BLASTN program (http://www.ncbi.nlm.nih.gov/), the sequences were found to have 100% similarity index with Lcs 16s RNA sequence. Lcs was cultured at 37 °C using MRS agar. The bacterial concentration was standardized at an optical density of 1.0 using UV–VIS spectrophotometer (UV-1800, Shimadzu, Kyoyo, Japan) at 600 nm wavelength. Using plate counting, the bacteria was then measured at 10^9^ cells in MRS agar [57]. The bacterial stock cultures were maintained at −20 °C in 10 % (*v*/*v*) glycerol after centrifugation (5417, Eppendorf, Barkhausenweg, Germany) at 3500 rpm for 15 min. Meanwhile, the working cultures were kept in MRS agar at 4 °C [58]. Prior to the oral administration, the glycerol liquid was removed and replaced with 200 μL PBS solution [59].

### 4.3. Experimental Animals

A total of twenty-four (*N* = 24) 7–8 weeks old Sprague Dawley (SD) rats (male, 250–300g) were used in this study [60]. The animals were supplied by Animal Resource Unit (ARU), Faculty of Veterinary Medicine, University Putra Malaysia (UPM). The protocol was carried out in the animal research house of Comparative Medicine and Technology Unit (COMeT), Institute of Bioscience UPM. The cages with wood shavings were used for housing the rats in groups of two or three. The rats are acclimatized for one week prior to the experiment under regulated temperature (20–22 °C), 12 h light-dark cycle (0700–1900 h), and feed on a normal diet and water *ad-libitum* [60]. The weight and feed intake of the rats in all groups were measured and monitored every week.

### 4.4. Experimental Protocol

The rats were separated into three different groups randomly (*n* = 8): Control, AFB1, and Lcs + AFB1. Control: Oral gavaged with 1× PBS buffer at pH 7.4; AFB1 group: Fed with AFB1 only via oral gavage; Lcs + AFB1 group: Supplemented daily with 10^9^ CFU Lcs by oral gavage. After five days of probiotic treatment, the rats were fed with AFB1 daily 4 h after Lcs treatment. The complete dosage given to the rats were 25 μg AFB1/kg body weight (b.w.) for five days per week [61]. The concentration of AFB1 fed on rats in the present study was chosen according to the AFB1 level (30–450 ng/mL) found in the diet of developing countries [62]. Throughout the experiment, the animals were given *ad libitum* access to food and water. The health status of rats was monitored every week. After a treatment period of four weeks, rats were subjected to anaesthesia using ketamine-xylazine.

### 4.5. Gut Microbiome Modulation via Administration of Lactobacillus casei Shirota on AFB1-Induced Rat

#### 4.5.1. Fecal Sample Collection

At the end of the study, the rats were kept in metabolic cages for feces collection [63]. The feces were sampled using sterile tweezers to avoid cross-contamination [63]. In order to protect the quality of the samples, the samples were kept at −80 °C.

#### 4.5.2. Extraction of Fecal Sample DNA

Based on the manufacturer’s protocol, the fecal samples were subjected to bacterial DNA extraction at the same time using fecal QiaAmp DNA Stool Mini Kit (Qiagen, Hilden, Germany) [64].

#### 4.5.3. Metagenomic Sequencing of Gut Microbiota

Metagenomic sequencing was performed based on the protocol used in a study by Kelly et al. [65]. The library preparations of next-generation sequencing and Illumina MiSeq sequencing were both performed at GENEWIZ, Inc. (Suzhou, China). The DNA samples extracted were measured using Qubit 2.0. Fluorometer (Invitrogen, Grand Island, NY, USA). Approximately 30-50 ng DNA was used to generate amplicons using a MetaVx™ Library Preparation kit (GENEWIZ, Inc., South Plainfield, NJ, USA). The hypervariable regions of V3 and V4 in bacterial 16S rDNA were amplified and subjected to subsequent taxonomic analysis. The conserved regions bordering the V3 and V4 regions were amplified using forward primers “CCTACGGRRBGCASCAGKVRVGAAT” and reverse primers “GGACTACNVGGGTWTCTAATCC”. Next, the products from the first polymerase chain reaction (PCR) were amplified in a second round of PCR. For the generation of indexed libraries, the indexed adapters were connected to the 16S rDNA amplicons for NGS sequencing steps using Illumina Miseq. The DNA libraries generated were validated using Agilent 2100 Bioanalyzer (Agilent Technologies, Santa Clara, CA, USA), and measured using Qubit 2.0 Fluorometer [66]. Next, the DNA libraries were multiplexed and inserted into an Illumina MiSeq instrument based on manufacturer’s instructions (Illumina, San Diego, CA, USA). Lastly, the sequencing was completed using a 2 × 300 paired-end (PE) configuration. MiSeq Control Software (MCS, 2.6.2, Illumina, Inc., San Diego, CA, USA, 2016) embedded in the MiSeq instrument carried out the image analysis and base calling [67].

#### 4.5.4. Data Analysis

The data analysis of 16S rRNA was performed using Quantitative Insight Into Microbial Ecology (QIIME) open-source software package version 1.9.1 [68]. Based on barcode, the forward and reverse reads were merged and designated to samples. The sequences were trimmed by removing the barcode and primer sequence. Following that, quality filtering on the joined sequences was conducted based on the following criteria: No ambiguous bases, sequence length <200 bp, mean quality score ≥ 20. A reference database (RDP Gold database) was used to detect chimeric sequence via UCHIME algorithm. The sequences that did not fulfill the criteria were removed, therefore the final analysis only involved the effective sequences.

Using the clustering program VSEARCH(1.9.6) against the Silva 119 database pre-clustered at 97% sequence identity, the sequences were clustered into OTUs. Next, all OTUs were assigned into taxonomic category up to the species level at a confidence threshold of 0.8 based on Silva 123 database using Ribosomal Database Program (RDP) classifier [69]. The phylogenetic tree of the top 30 OTU sequences was plotted using R software version 3.3.1 (https://www.r-project.org/, Lucent Technologies, Inc., Murray Hill, NJ, USA, 2014) [70]. The core gut microbiota’s Venn diagram (OTU overlapping) was generated by the VennDiagram package in R software [70]. The sequences were subjected to the alpha and beta diversity analysis in QIIME after rarefying steps. Alpha diversity indexes include the richness indicated by Chao1 index and the for diversity indicated by Shannon index [33]. On the other hand, beta diversity was evaluated from weighted and unweighted UniFrac, PCA, NMDS, and clustered using UPGMA [36,71].

### 4.6. Statistical Analysis

Each sample was analyzed with four replicates for three different group combinations (Control vs AFB1; Control vs Lcs + AFB1; and AFB1 vs Lcs + AFB1). The statistical difference between-groups vs the difference within-group was analyzed by ANOSIM analysis using R software [40]. For the variation analysis between groups, LEfSe (LDA Effect Size) was performed to reveal taxonomic characteristics and to characterize the differences between the two groups [42]. LEfSE was performed using online available software Galaxy version 1.0 (http://galaxyproject.org/) [72] and STAMP version 2.1.3. Significance difference was determined based on *p*-value < 0.05.

## Figures and Tables

**Table 1:** Group difference evaluation by analysis of similarities (ANOSIM).

Factor	*R*-Value	*p*-Value
Control vs AFB1	1	0.025
Control vs Lcs + AFB1	0.292	0.094
AFB1 vs Lcs + AFB1	0.87	0.025

Note: The value of *R* ranges from 0 to 1. The closer it is to 0, the less significant the between-group difference is compared to within-group difference; the closer it is to 1, the more significance the between-group difference compared to within-group difference.

## Supplementary Materials

The following are available online at http://www.mdpi.com/2072-6651/11/1/49/s1, Figure S1: Cladograms that indicate the evolutionary relationships of different species between the two groups (AFB1 group vs Control group, A; Control group vs Lcs + AFB1 group, B; AFB1 groupvs Lcs + AFB1 group, C).
