# Supplementary material for: Gut Microbiota Profiling of Aflatoxin B1-Induced Rats Treated with Lactobacillus casei Shirota
Source: Toxins (Basel). 2019 Jan 17;11(1):49. doi: 10.3390/toxins11010049 (PMC6357033; doi:10.3390/toxins11010049)
Supplement: Supplementary file 1 [file toxins-11-00049-s001.pdf]

Toxins **2019**, *11*, 49 www.mdpi.com/journal/toxins

Supplementary Materials: Gut Microbiota Profiling of Aflatoxin B1-Induced Rats Treated with *Lactobacillus casei* Shirota

Winnie-Pui-Pui Liew, Sabran Mohd-Redzwan and Leslie Thian Lung Than

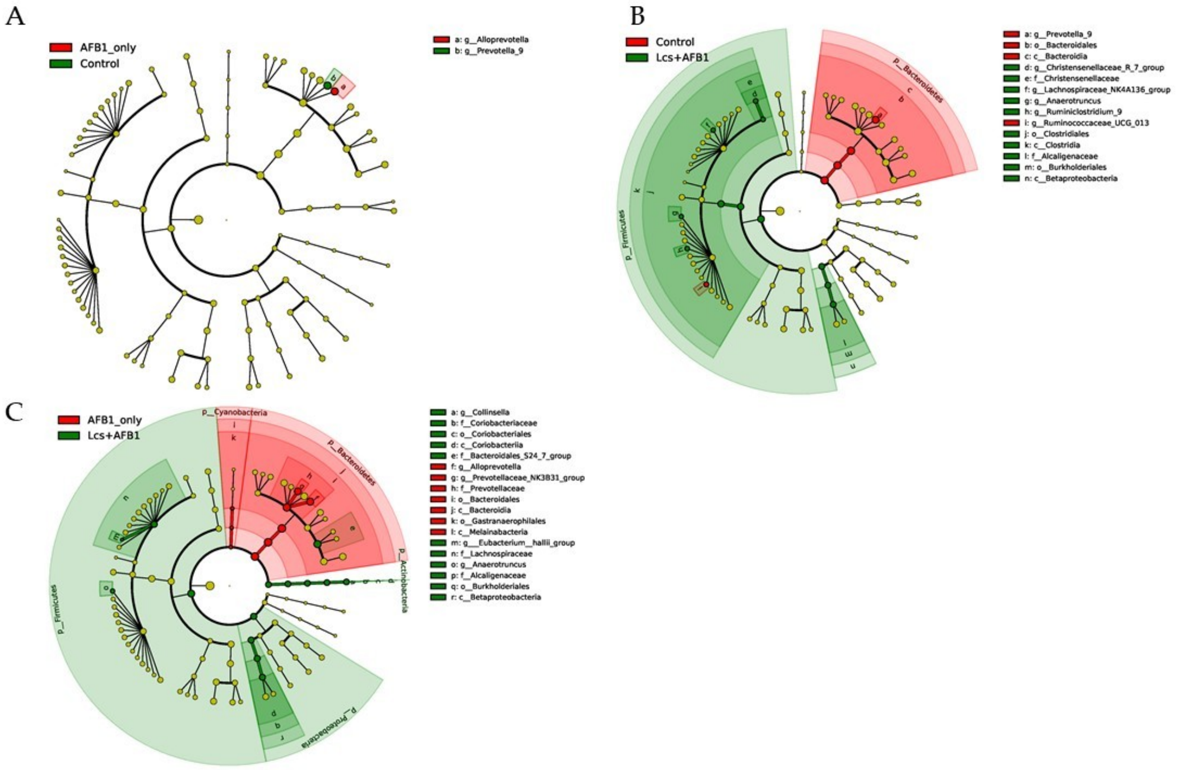

Figure S1. Cladograms that indicate the evolutionary relationships of different species between the two groups (AFB1 group vs Control group, (**A**) Control group vs Lcs + AFB1 group, (**B**) AFB1 group vs. Lcs + AFB1 group, (**C**). Nodes of different background colors (red or green) indicates different groups. The nodes in red represent microorganism groups that play an important role in the red group; the nodes in green represents microorganism groups that play an important role in the green group; the nodes in yellow represents the groups that do not play an important role in either group. The names of the species represented by the letters in the figure are shown on the right.
